# Association of body roundness index and its trajectories with all-cause and cardiovascular mortality among a Chinese middle-aged and older population: A retrospective cohort study

**DOI:** 10.3389/fpubh.2023.1107158

**Published:** 2023-03-23

**Authors:** Jiacheng Ding, Xuejiao Chen, Zhan Shi, Kaizhi Bai, Songhe Shi

**Affiliations:** ^1^Department of Epidemiology and Health Statistics, College of Public Health, Zhengzhou University, Zhengzhou, Henan, China; ^2^Department of Pharmacy, Zhengzhou People’s Hospital, Zhengzhou, Henan, China

**Keywords:** cardiovascular risk, body roundness index, trajectories, retrospective cohort study, obesity

## Abstract

**Objectives:**

The body roundness index (BRI) is a novel anthropometric index that is a better indicator for predicting fat distribution than the body mass index (BMI). The longitudinal study can repeatedly collect measured results for the variables to be studied and then consider the potential effects of intraindividual changes in measurement. However, few population-based, longitudinal studies of BRI have been conducted, especially among the Chinese population. The study aimed to investigate the association of BRI and its longitudinal trajectories with all-cause and cardiovascular mortality.

**Methods:**

A total of 71,166 participants with four times BRI measurements between January 2010 and December 2019 were included in this longitudinal study, with a median follow-up was 7.93 years, and 11,538 deaths were recorded, of which 5,892 deaths were due to cardiovascular disease (CVD). A latent class growth mixture modeling (LCGMM) was used to identify BRI trajectories. Cox proportional hazard models were used to estimate associations between BRI trajectories and the risk of all-cause and cardiovascular mortality.

**Results:**

In the restricted cubic spline regression models, a U-shaped relationship between BRI and all-cause and cardiovascular mortality was observed. Three BRI longitudinal trajectories of low-stable (mean BRI = 2.59), moderate-stable (mean BRI = 3.30), and high-stable (mean BRI = 3.65) were identified by LCGMM. After being adjusted for potential confounders, the HRs for all-cause mortality were 1.18 (1.13–1.24) for the moderate-stable group and 1.74 (1.66–1.82) for the high-stable group compared to the low-stable group. The HRs for cardiovascular mortality were 1.12 (1.05–1.18) for the moderate-stable group and 1.64 (1.53–1.75) for the high-stable group compared to the low-stable group.

**Conclusion:**

A nonlinear association of BRI with all-cause and cardiovascular mortality was observed, and participants in the higher BRI longitudinal trajectory group were significantly associated with an increased risk of all-cause and cardiovascular mortality.

## Introduction

With a rapidly aging global population and epidemiologic changes in disease, cardiovascular disease remains a significant cause of both morbidity and mortality globally, especially for middle-aged and older adults (1, 2), which also causes a substantial economic burden on society (3, 4). Obesity is a well-known independent risk factor for CVD and mortality (5). Most previous work has focused on the relationship between BMI with CVD and mortality (5, 6). However, studies in recent years have revealed that because BMI is not sufficient to distinguish between muscle and fat mass, it may not adequately reflect the fat distribution (7–9), mainly when abdominal fat is strongly associated with CVD (10, 11). As a complement to BMI, the BRI, which combines height and waist circumference (WC) measurements, is a new anthropometric index that is a better indicator to describe fat distribution (12).

Prior studies have suggested that BRI is a valuable predictor of cardiovascular disease in men and women in the Chinese population, but most of them are based on a cross-sectional design (13, 14). The longitudinal study design provides the opportunity to collect measured results for the variables to be studied repeatedly and then take the potential effects of intraindividual changes in measurement into account (15–17). Group-based trajectory modeling techniques such as LCGMM are a universal approach to illustrate the development of the variable over time and can be used to disentangle underlying population heterogeneity (18, 19). However, very few studies were conducted on the longitudinal trajectory of BRI, especially among the middle-aged and older Chinese population. A study focused on trajectories of BMI prior to CVD diagnosis identified three distinct BMI trajectories, probably due to the small number of study participants(*n* = 6,126), and the results suggested that BMI alone is not sufficient to identify people at high risk for CVD in middle-aged and older adults (20). Besides, a recent study based on a longitudinal trajectory model found that higher levels of BRI over time were statistically related to a higher risk of CVD and mortality. However, this study seemed to be restricted by a short follow-up time and almost a 4:1 male-to-female ratio (21). Therefore, our study aimed to examine the association of BRI with all-cause and cardiovascular mortality, identify longitudinal trajectories in BRI, and then estimate the associations of BRI trajectories with all-cause and cardiovascular mortality based on a sizeable dynamical cohort study.

## Method

### Study population

This retrospective cohort study was performed in a dynamic population based on an annual health check-up project, and it has been carried out since 2010 in Xin Zheng, Henan Province. All participants were asked to complete a questionnaire and to take anthropometric and laboratory measurements at baseline and follow-up. Details of this dynamic cohort have been described previously (22–24). The data were analyzed from residents’ electronic health records in the Xin Zheng Hospital Information System from January 2010 to December 2019. To ensure the quality of the cohort and trajectories, the records with missing data for BRI were removed, and each study participant kept one health examination record per year. Between January 2010 and December 2019, 102,797 participants with four or more medical records were enrolled. We excluded 31,631 individuals who met with any one of the following circumstances: a history of CVD at baseline (*n* = 18,704); aged less than 45 years old (*n* = 7,981); missing information (*n* = 1,682) on BMI at baseline; missing information (*n* = 3,264) on smoking, drinking, physical activity, and Marital status at baseline. The records and times of the four examinations of the participants are presented in Supplemental Table S1. Finally, in the study, we have data from 71,166 participants with four recordings, including when the outcome occurred.

### Data collection

Data were collected through a standardized questionnaire, as well as from physical and laboratory examinations. Standardized questionnaire of the National Norms for Basic Public Health Services (Third Edition), which included their sociodemographic characteristics (age, sex), medical history (coronary heart disease (CHD) and stroke), smoking, drinking, and physical activity, were administered by trained research staff. Based on self-reported marital status, smoking, and drinking, participants were classified as follows: living with a partner or without a partner; nonsmokers (including previous smokers) or current smokers; and never, occasionally, or daily drinkers. The frequency of physical activity was described as never, occasionally, and daily (25).

Standing height and weight were measured to the nearest 0.1 cm and 0.1 kg with the participant standing erect on bare feet, and the results were recorded by the mean of two replicate measurements. BMI was calculated as weight (kg) divided by height squared (m). Waist circumference was measured to the nearest 0.1 cm at the midpoint between the lowest rib margin and the iliac crest following a standard protocol. Blood pressure was measured at least twice using an automatic sphygmomanometer (OMRON HEM-7125, Kyoto, Japan), and the mean of the two measurements qualified was used in the analysis.

### Assessment of BRI

BRI was calculated as follows: (12).

$BRI=364.2-365.5\times \sqrt{1-\frac{{\left(WC/2\pi \right)}^{2}}{{\left(0.5height\right)}^{2}}}$, and then the quartiles of BRI and its trajectories were used to statistical analysis.

### Assessment of outcomes

The primary outcomes in the study were all-cause and CVD mortality, where CVD death was defined as death from CHD and death from stroke. For mortality surveillance, participants’ mortality information was obtained from the Xinzheng Center for Disease Control and Prevention from the baseline survey to October 7, 2022. The causes of death were recorded using codes from the International Classification of Diseases (ICD-10), in which death from CVD was defined as I20eI25 and I60eI69.

### Statistical analyzes

For non-normal distribution, continuous variables are characterized by the median (interquartile range (IQR)), while categorical variables are expressed as frequency (%). The Kruskal-Wallis test was used to compare continuous variables and the Chi-square test for categorical variables.

### Latent BRI trajectories identification

The latent class growth mixture modeling (LCGMM) was used to explore heterogeneity in the dynamic course of BRI to distinguish subgroups of similar underlying BRI trajectories as experienced over time. Models were fit using the package “LCMM” (version 2.0.0.) in R to group participants with a similar trajectory of BRI development from the first examination to the fourth (18). Three possible polynomial specifications were allowed to describe the longitudinal BRI response as a function of time: a linear, quadratic, and a cubic specification, and every polynomial model (order 1 to 3) was, respectively, modeled as a 1 to 4 class solution. Given that no clear standard exists, the choice of the best model was evaluated by the following composite criteria: (1) observing improvement in the Bayesian information criterion (BIC); (2) at least 5% participants in each trajectory class; (3) values of mean posterior class membership probabilities (>70%); (4) confirming visually distinct trajectories. (21, 26) For ease of interpretation, we have assigned labels to these trajectories based on their modeled graphic patterns.

Cox proportional hazards models were used to estimate hazard ratios (HRs) and 95% confidence intervals (CIs) between all-cause and cardiovascular mortality and BRI trajectories, quartiles of BRI at baseline, and per 1sd increment in BRI after inspection of Schoenfeld residuals. Model 1 was unadjusted; Model 2 was adjusted for sex and age; Model 3 was adjusted for sex, age, smoking status, alcohol drinking level, and physical activity. We performed tests for linear trends by modeling BRI quartiles as ordinal variables. To assess nonlinearity, we performed a restricted cubic spline to the multivariable cox proportional hazards models, and then the cut-off value was estimated by trying all possible values and choosing the cut-off point with the highest likelihood. We performed subgroup analyzes based on sex. To verify the robustness of the results, we conducted an additional sensitivity analysis, excluding those participants with less than 3 years of follow-up. Besides, we similarly analyzed the trajectories of BMI and waist circumference of the participants in our study.

*p* < 0.05 for a two-sided test was regarded as statistically significant. All analyzes were performed using R version 4.1.3 (R Foundation for Statistical Computing).

## Results

### Baseline characteristics

The baseline characteristics of the study sample, stratified by all-cause and cardiovascular mortality, are summarized in Table 1. A total of 71,166 study participants (women: 36,503) were included in the present study. The median age (interquartile range) for women and men was 61.75 (56.78–69.13) and 61.97 (57.38–68.56), respectively. During the 512,131 person-years of follow-up (median follow-up time 7.93 years), 11,538 deaths were recorded, of which 5,892 deaths were due to CVD, and 4,065 and 2,108 cases of CHD and stroke, respectively. Compared with participants who survived to the end of the study, the decedents were older, were more likely to be male, living with a partner, and had a higher proportion of smokers. Similar demographic characteristics were observed in participants who died from cardiovascular disease, except for the proportion of smoking.

**Table 1:** Baseline characteristics of the study population stratified by outcome.

Variables	All-cause mortality		*p* value	Cardiovascular disease mortality		*P* value
	No (*n* = 59,628)	Yes (*n* = 11,538)		No (*n* = 65,274)	Yes (*n* = 5,892)	
Age (years)	60.75 (56.48, 66.11)	71.81 (63.99, 77.78)	<0.001	61.31 (56.76, 67.56)	71.95 (64.28, 77.85)	<0.001
Gender (%)			<0.001			<0.001
Women	31,366 (52.60)	5,137 (44.52)		33,781 (51.75)	2,722 (46.20)	
Men	28,262 (47.40)	6,401 (55.48)		31,493 (48.25)	3,170 (53.80)	
Marital status (%)			<0.001			<0.001
Living without partner	8,767 (14.70)	3,906 (33.85)		10,591 (16.23)	2,082 (35.34)	
Living with partner	50,861 (85.30)	7,632 (66.15)		54,683 (83.77)	3,810 (64.66)	
Smoking (%)			0.0075			0.1496
Never or previous	52,055 (87.30)	9,967 (86.38)		56,923 (87.21)	5,099 (86.54)	
Current	7,573 (12.70)	1,571 (13.62)		8,351 (12.79)	793 (13.46)	
Drinking (%)			<0.001			<0.001
Never	55,271 (92.69)	10,702 (92.75)		60,539 (92.75)	5,434 (92.23)	
Occasionally	3,513 (5.89)	566 (4.91)		3,781 (5.79)	298 (5.06)	
Daily	844 (1.42)	270 (2.34)		954 (1.46)	160 (2.72)	
Physical activity (%)			<0.001			0.0015
Never	48,389 (81.15)	9,584 (83.06)		5,3,107 (81.36)	4,866 (82.59)	
Occasionally	5,212 (8.74)	1,016 (8.81)		5,700 (8.73)	528 (8.96)	
Daily	6,027 (10.11)	938 (8.13)		6,467 (9.91)	498 (8.45)	
WC	80.00 (75.00, 85.32)	80.00 (74.70, 85.00)	<0.001	80.00 (75.00, 85.00)	80.00 (74.00, 85.00)	<0.001
BMI	23.66 (22.23, 25.54)	23.19 (21.48, 24.97)	<0.001	23.62 (22.20, 25.46)	23.31 (21.60, 25.15)	<0.001
BRI	3.25 (2.72, 3.88)	3.14 (2.62, 3.78)	<0.001	3.24 (2.71, 3.87)	3.19 (2.62, 3.83)	<0.001
Time of follow-up	8.08 (6.08, 8.93)	5.94 (4.07, 7.36)	<0.001	8.00 (5.96, 8.92)	5.99 (4.17, 7.81)	<0.001

Data are presented, median (interquartile range), or number (percentage). BMI, body mass index; WC, waist circumference; BRI, body roundness index.

### Associations of BRI and its trajectories with all-cause and cardiovascular mortality

**Figure 1:**
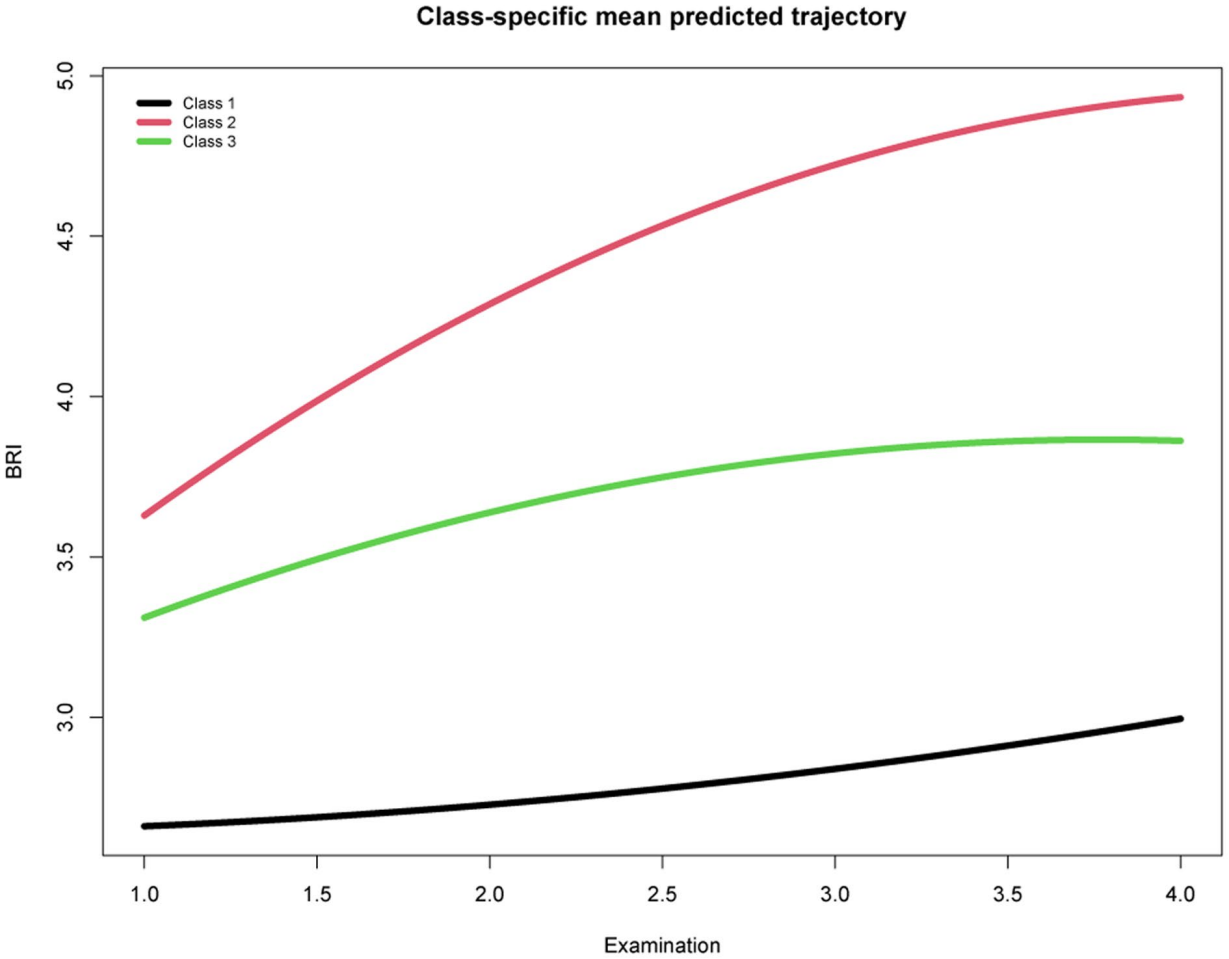
Trajectories of body roundness index from the first physical examination to the fourth.

Based on the BIC, class membership posterior probabilities, and classification to assess the goodness-of-fit of the competing LCGMM models, the model with 3 BRI trajectories among the 71,166 participants was identified as the best-fit model: there were low-stable (mean BRI = 2.59, *n* = 12,972), moderate-stable (mean BRI = 3.30, *n* = 26,796), and high-stable (mean BRI = 3.65, *n* = 31,398) trajectories (Supplemental Tables S2, S3; Figure 1). Though several identified latent classes had a low proportion of participants (<20%), they were highly discriminated with high mean posterior probabilities and posterior probabilities. The proportion of men was higher in the “high-stable” and “moderate-stable” trajectory groups, but the “low-stable” group contained more women. Compared with participants in the low-stable class, counterparts in the other two groups were more likely in higher BMI and waist circumference values.

**Figure 2:**
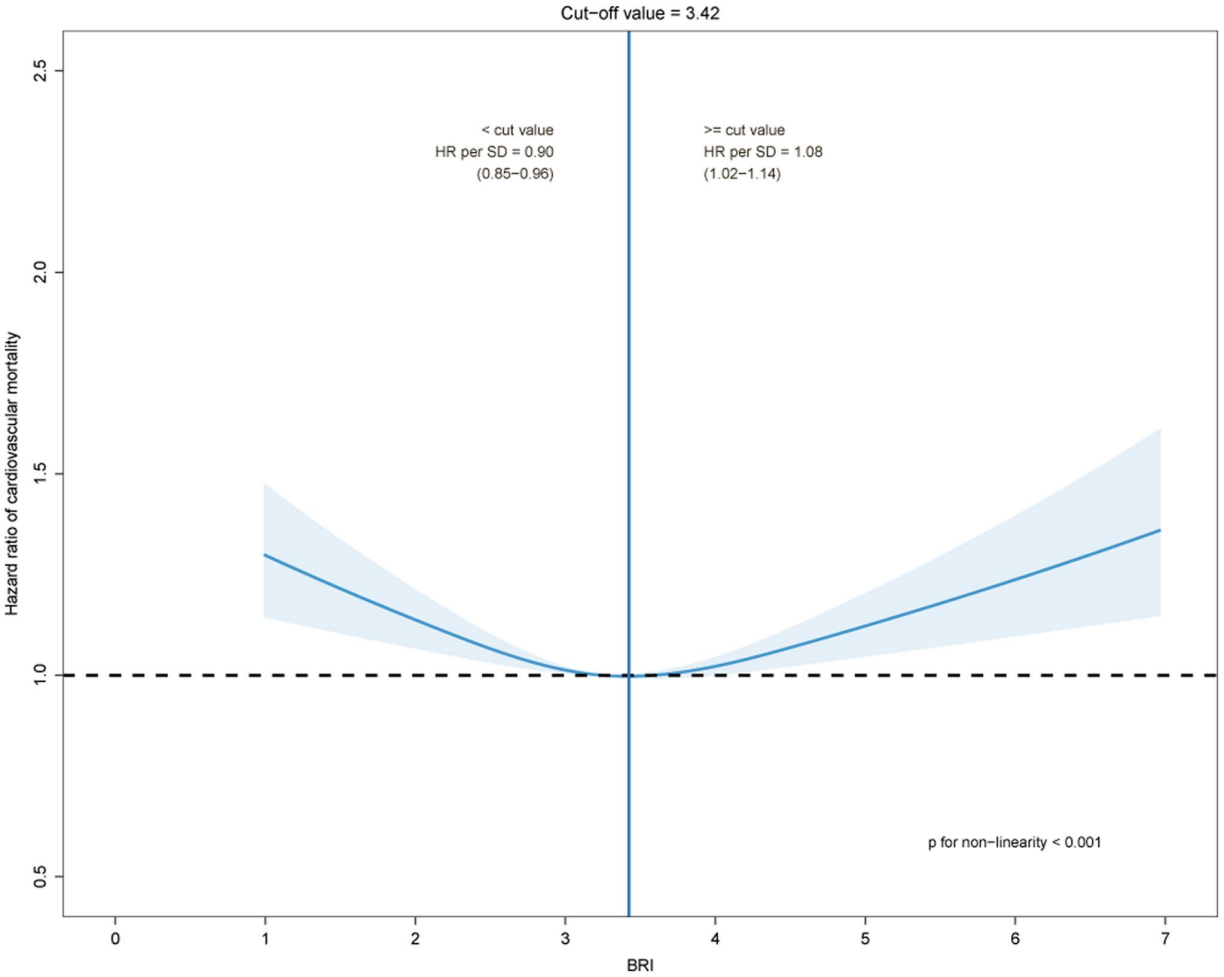
The dose–response relationships between the body roundness index and cardiovascular mortality by restricted cubic spline models.

**Figure 3:**
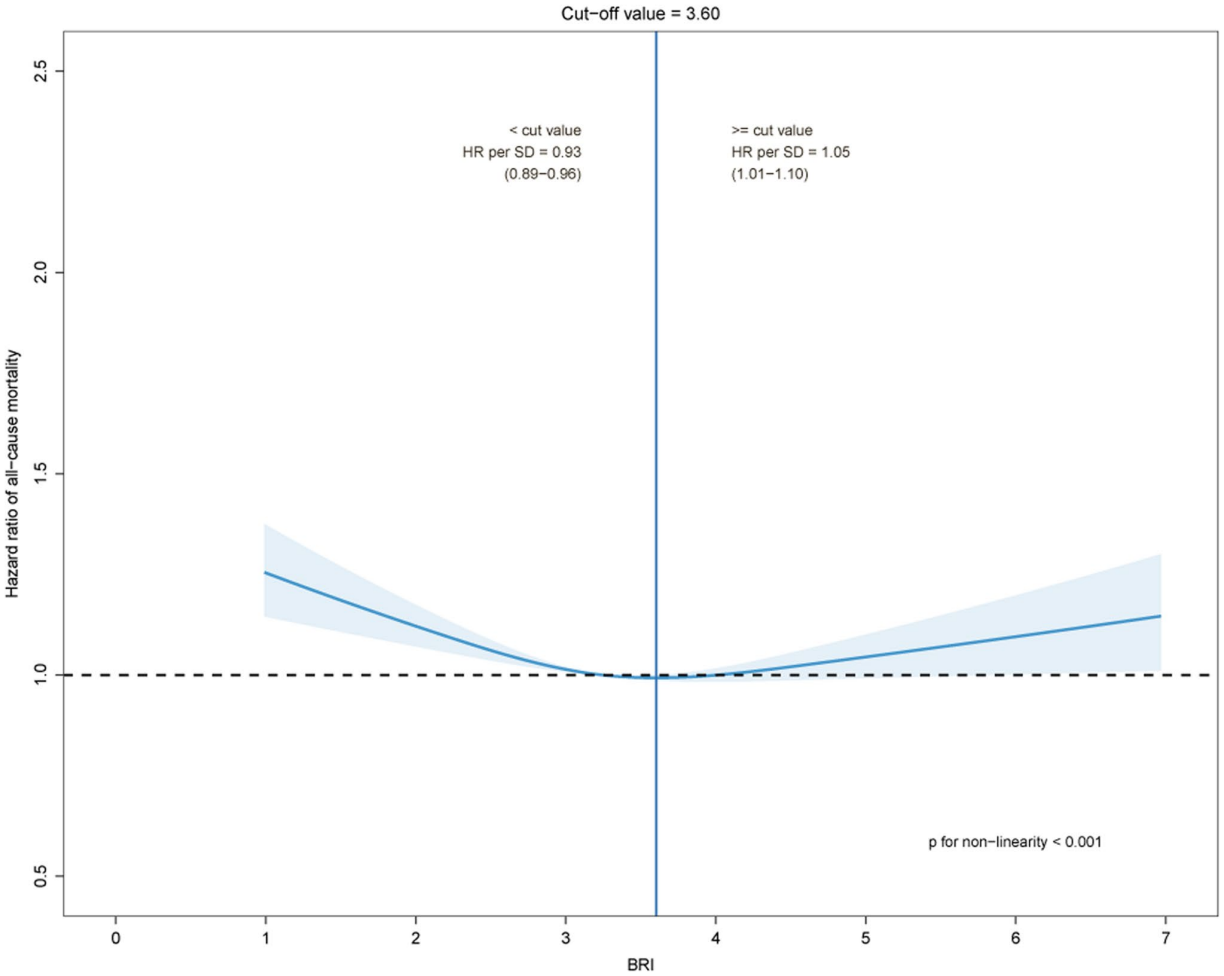
The dose–response relationships between the body roundness index and all-cause mortality by restricted cubic spline models.

The estimated risk for all-cause and cardiovascular mortality by BRI quartile and its trajectories were presented in Table 2. After adjusting for potential confounders, as compared with quartile 1 of BRI, with quartiles 2, 3, and 4, the HRs for all-cause mortality were 0.96(0.92–1.01), 0.91(0.86–0.96) and 0.95(0.89–0.99), respectively (*P* trend =0.010); the HRs for cardiovascular mortality were 0.92(0.86–0.98), 0.93(0.87–0.99) and 0.98(0.91–1.05), respectively (*P* trend =0.617). Further, compared with the low-stable category, counterparts in the other two groups had a significantly higher risk of all-cause and CVD mortality. After being adjusted for potential confounders, the HRs for all-cause mortality were 1.18 (1.13–1.24) for the moderate-stable group and 1.74 (1.66–1.82) for the high-stable group. The HRs for cardiovascular mortality were 1.12 (1.05–1.18) for the moderate-stable group and 1.64 (1.53–1.75) for the high-stable group. The dose–response relationships between the BRI and all-cause and cardiovascular mortality by restricted cubic spline models in the middle-aged and elderly population were presented in Figures 2, 3. The cut-off values of all-cause and cardiovascular mortality were 3.60 and 3.42, respectively, and while BRI < 3.60, the HR for all-cause mortality was 0.93 (0.89–0.96) as per 1 SD BRI increased, *p* < 0.001. As BRI was more than 3.60, the HR for all-cause mortality was 1.05 (1.01–1.10) as per 1 SD BRI increased, *p* = 0.026. Similar results were observed for cardiovascular disease mortality.

**Table 2:** Cox regression analysis between BRI and all-cause mortality and cardiovascular mortality.

		Model 1				Model 2			Model 3	
		HR	95%CI	*P*	HR	95%CI	*P*	HR	95%CI	*P*
**All-cause mortality**									
Q1(2.03–2.54)	1(Ref)			1(Ref)			1(Ref)		
Q2(2.82–3.11)	0.87	0.83, 0.92	<0.001	0.97	0.92, 1.02	0.214	0.96	0.92, 1.01	0.158
Q3(3.36–3.67)	0.79	0.75, 0.84	<0.001	0.91	0.86, 0.96	<0.001	0.91	0.86, 0.96	<0.001
Q4(4.09–4.91)	0.87	0.83, 0.92	<0.001	0.95	0.90, 0.99	0.036	0.95	0.89, 0.99	0.039
*P_trend_*			<0.001			0.008			0.010
Low-stable	1(Ref)			1(Ref)			1(Ref)		
Moderate-stable	1.17	1.12, 1.23	<0.001	1.18	1.13, 1.23	<0.001	1.18	1.13, 1.24	<0.001
High-stable	2.09	1.99, 2.19	<0.001	1.74	1.66, 1.82	<0.001	1.74	1.66, 1.82	<0.001
**CVD mortality**									
Q1(2.03–2.54)	1(Ref)			1(Ref)			1(Ref)		
Q2(2.82–3.11)	0.83	0.77, 0.89	<0.001	0.92	0.86, 0.99	0.027	0.92	0.86, 0.98	0.018
Q3(3.36–3.67)	0.81	0.76, 0.87	<0.001	0.93	0.87, 1.01	0.056	0.93	0.87, 0.99	0.043
Q4(4.09–4.91)	0.91	0.84, 0.97	0.006	0.98	0.91, 1.05	0.597	0.98	0.91, 1.05	0.532
*P_trend_*			0.009			0.675			0.617
Low-stable	1(Ref)			1(Ref)			1(Ref)		
Moderate-stable	1.09	1.03, 1.16	0.004	1.11	1.05, 1.18	<0.001	1.12	1.05, 1.18	<0.001
High-stable	1.94	1.82, 2.07	<0.001	1.64	1.53, 1.75	<0.001	1.64	1.53, 1.75	<0.001

Data were showed by HR, 95% CI and *P* value. Model 1: Unadjusted. Model 2: Adjusted for age and gender. Model 3: Adjusted for age, gender, smoking, alcohol consumption, and physical activity. Q1: 1st Quartiles; Q2: 2nd Quartiles; Q3: 3rd Quartiles; Q4: 4th Quartiles. Q1 to Q4 are the quartiles of BRI at baseline.

### Subgroup analyzes and sensitivity analyzes

The results of the subgroup analysis by sex were generally consistent with the primary analysis in Supplemental Figures S1, S2. In all-cause and cardiovascular mortality, there was a U-shaped relationship for both sexes, but differences in cutoff values emerged. For cardiovascular mortality, the BRI cutoff value was higher in women than in men, while in all-cause mortality, it was higher in men than in women. Furthermore, a sensitivity analysis showed similar results to the primary analysis. When excluding those participants with less than 3 years of follow-up, the parameters of the LCGMM model were also much optimized, and similar results of the association of BRI and its trajectories with all-cause and cardiovascular mortality were observed in Supplementary Tables S4, S5. From Supplementary Figures S3, S4, it can be found that both BMI and WC trajectories appear crossed and do not have a good differentiation ability compared to BRI, and the details of the trajectory models of BMI and WC are presented in Supplementary Tables S6, S7. Besides, after comparing the results of the Cox regression models, it was found that only the results of BRI are statistically significant. (Supplementary Tables S8, S9).

## Discussion

The median follow-up of the study was 7.93 years (range 5.86–8.92), with four examinations in all participants. A U-shaped trend in the association between BRI and all-cause and cardiovascular mortality was observed in the study. The all-cause and cardiovascular mortality were lowest when the BRI was 3.60 and 3.42, respectively. During the follow-up period, according to the BRI trajectory of the study participants, we divided them into three groups, namely, low-stable, moderate-stable, and high-stable. Overall, the BRI of the study subjects showed a steady ascending trend during the entire study follow-up.

As a universal indicator of obesity, BMI has always been a hot spot for research. Several epidemiological studies have found a U-shaped association between BMI and all-cause and cardiovascular mortality, including in the middle-aged and elderly population. (27–29) At the same time, a J-shaped association has also been found between BMI and all-cause and cardiovascular mortality. (28, 30) Previous research suggested that this debate may lie in the fact that BMI does not discriminate well between fat mass and lean body mass. (31) Meanwhile, an association between BRI and cardiovascular mortality was observed (13, 32, 33), including a U-shaped relationship (34), especially when BRI was able to better distinguish the proportion between total fat and abdominal fat. (12) The proliferation and hypertrophy of adipocytes can affect the integrity and functionality of adipose tissue, which further affects the risk of cardio metabolism. (35, 36) Adipose tissue causes damage to cardio metabolism by affecting metabolic homeostasis, which in turn creates a state of low-grade systemic inflammation and insulin resistance. (37) Furthermore, the expansion of adipose tissue can cause a series of bodily reactions, such as fibrosis (38), hypoxia (39), inflammation (40), dysregulated adipokine secretion (41), a pro-inflammatory and pro-thrombotic state and endothelial dysfunction, all of which would be cardiovascular risk factors. (42) Especially for the middle-aged and elderly population, BRI was related to malnutrition, fatigue, decreased activity tolerance, and muscle atrophy, all of these were risk factors for death in the elderly. (43, 44)

Although previous studies were abundant, most classified variables into predefined categories, such as based on established criteria or quartiles. It has been reported that this classification method may lead to the misclassification of those individuals close to the classification cutoff point. (20) Whereas, in the LGCMM model in this study, it was assumed that there was no single developmental curve in the study population and that individuals belonged to different subgroups with different developmental trajectories. The pattern of BRI changes during the follow-up period was modeled based on population heterogeneity. In recent years, many studies on the trajectory analysis of obesity indicators and cardiovascular disease have been reported. For instance, Marie-Jeanne Buscot (2018) and Kim Blond (2020) have investigated the association between distinct BMI trajectories in childhood and cardiovascular risk factors in adulthood. (18, 45) By analyzing BMI trajectories, Klodian Dhana et al. found that BMI alone is insufficient to identify high-risk subjects for cardiovascular disease in the middle-aged and elderly population. (20) A recent study of BRI trajectories conducted by Wu et al. found that higher BRI levels were significantly associated with an increased risk of CVD over time. (21) Our conclusions are consistent with these findings. In our middle-aged and elderly cohort, we identified three BRI trajectories that slowly increased over time and found that higher BRI levels were significantly associated with an increased risk of cardiovascular mortality.

Few studies, based on a large population, have assessed the association between BRI trajectories and cardiovascular disease risk. In fact, our study has important implications for the primary prevention of cardiovascular mortality in the middle-aged and elderly and public health. First, because of the U-shaped trend in the association between BRI and all-cause and cardiovascular mortality, from an individual level, maintaining the BRI at around 3.30 has the lowest risk. It is noteworthy that although no U-shaped association has been observed in waist circumference, it is also an important indicator of obesity signs. If more evidence becomes available in the future, it would be helpful for people to choose an optimal control range when managing body shape. Second, recent studies have revealed that the cardiovascular risk of obesity is cumulative and that the duration of obesity may substantially impact CVD outcomes. (18, 46) When participants with less than 3 years of follow-up were excluded, the model was enhanced, though the results remained robust, suggesting that the long-term patterns of BRI may have a potential additional impact on all-cause and cardiovascular mortality. Considering that cardiovascular disease prevention is long-term and dynamic, especially with increasing age and accumulation of co-morbidities, based on population considerations, our results emphasize that dynamic surveillance and multi-level prevention should be implemented on a long-term or even lifetime basis.

Strengths of this study include the cohort study design, the availability of repeated measurements of BRI, and identify groups of individuals with similar patterns of BRI trajectories based on long-term follow-up and repeated measurement. On the other hand, several limitations of the study are worth mentioning for future improvements. First, the study was conducted among middle-aged and elderly Chinese individuals with an average age of approximately 63.06 years, making it difficult to generalize to all populations. Second, although we have adjusted for some confounders as far as possible, the possibility of bias still existed, such as the use of antidiabetic, antihypertension drugs and other medications, dietary factors, genetic factors, and unavoidable recall bias. Then, only four physical examination records were kept for each participant during the follow-up period, which may have limited the ability to investigate potential effects.

## Conclusion

Overall, as an easily accessible anthropometric indicator of obesity, a U-shaped association between BRI and all-cause and cardiovascular mortality was observed in the study. In addition, the long-term trajectory study found that higher BRI levels were associated with an increased risk of all-cause and cardiovascular mortality over time, while there may be some potential effects of long-term patterns of BRI. Longitudinal trajectory models made up for some of the limitations of cross-sectional studies, but in the future, the potential impact caused by follow-up time needs to be examined further.

### Sharing statement of data

Due to third-party requirements for confidentiality, the raw data in the study are not currently available to the public but can be requested from the corresponding authors upon reasonable request.

## Data availability statement

The data analyzed in this study is subject to the following licenses/restrictions: Due to third-party requirements for confidentiality, the raw data in the study are not currently available to the public but can be requested from the corresponding authors upon reasonable request. Requests to access these datasets should be directed to Songhe Shi, ssh@zzu.edu.cn

## Ethics statement

Study procedures were performed in accordance with the Declaration of Helsinki ethical principles for medical research involving human subjects. The study was approved by the Ethics Committee of Zhengzhou University, and written informed consent was obtained from all participants (Reference Number: ZZUIRB2019-019).

## Author contributions

JD performed primary research design, statistical analysis, and wrote first draft. KB, ZS, and XC contributed to the analysis and interpretation of the data. SS planned overall, supervised the data analysis, and revised it critically. All authors approved the final version submitted and published and are responsible for all aspects of the study.

## Funding

This study was supported by the National Key Research and Development Program “Research on prevention and control of major chronic non-communicable diseases” of China. Grant number: 2017YFC130770.

## Conflict of interest

The authors declare that the research was conducted in the absence of any commercial or financial relationships that could be construed as a potential conflict of interest.

## Publisher’s note

All claims expressed in this article are solely those of the authors and do not necessarily represent those of their affiliated organizations, or those of the publisher, the editors and the reviewers. Any product that may be evaluated in this article, or claim that may be made by its manufacturer, is not guaranteed or endorsed by the publisher.
