# Supplementary material for: Association of body roundness index and its trajectories with all-cause and cardiovascular mortality among a Chinese middle-aged and older population: A retrospective cohort study
Source: Front Public Health. 2023 Mar 23;11:1107158. doi: 10.3389/fpubh.2023.1107158 (PMC10076882; doi:10.3389/fpubh.2023.1107158)
Supplement: Supplementary file 1 [file Data_Sheet_1.PDF]

Supplementary Material

Association of body roundness index and its trajectories with all-cause and cardiovascular mortality among a Chinese middle-aged and older population: a retrospective cohort study

Jiacheng Ding1, Xuejiao Chen1, Zhan Shi2, Kaizhi Bai1, Songhe Shi1*

*** Correspondence:**

Songhe Shi

ssh@zzu.edu.cn

Supplementary Table 1 The records and times of the four examinations

	2010	2011	2012	2013	2014	2015	2016	2017	2018	2019
1th examination	65376	8177	5187	2490	1429	871	563	NA	NA	NA
2th examination	NA	9566	7232	10246	4997	9257	9041	33754	NA	NA
3th examination	NA	NA	7240	7843	6849	6166	5377	11295	39323	NA
4th examination	NA	NA	NA	2931	3283	6045	5971	7949	9270	48644

Supplementary Table 2 Latent Class Growth Mixture models (LCGMM) results

Nb. Latent classes	Polynomial degree	Log-Lik	BIC	% Participants per class	Mean posterior probabilities	Posterior probabilities>70 (%)
1	Linear	-392290.7	784648.3	100	n.a	n.a
	Quadratic	-387521.4	775154.4	100	n.a	n.a
	Cubic	-386349.9	772867.4	100	n.a	n.a
2	Linear	-390998.7	782109.2	48.77/51.23	0.70/0.81	54.73/69.85
	Quadratic	-384474.6	769116.9	46.64/53.36	0.73/ 0.85	63.62/76.86
	Cubic	-382962.7	766160.1	45.91/54.09	0.72/ 0.85	62.65/77.06
3	Linear	-390838.4	781833.3	39.75/25.7/34.55	0.68/0.71/0.51	47.37/49.83/0
	Quadratic	-382868.7	765960.9	18.23/44.12/37.65	0.76/0.86/0.67	64.56/79.69/46.22
	**Cubic**	**-381244**	**762789.7**	**16.27/75.94/7.79**	**0.81/0.89/0.79**	**71.12/88.23/66.80**
4	Linear	-391000.1	782201.4	49.62/0/21.01/29.37	0.52/n.a/0.49/0.33	0/n.a/8.47/0
	Quadratic	-382307.6	764894.6	7.21/30.62/35.53/26.64	0.78/0.69/0.81/0.60	66.41/54.56/70.63/17.55
	Cubic	-385305.5	770979.6	0/45.44/54.56/0	n.a /0.73/0.84/n.a	n.a/62.79/76.25/n.a

Supplementary Table 3 Baseline characteristics of participants according to the trajectories of BRI

Characteristics	Low-stable	Moderate-stable	High-stable	P
Num	12,972	26,796	31,398	
Age (years)	63.25 (57.44, 71.79)	61.37 (56.63, 68.08)	61.82 (57.27, 68.41)	<0.001
Gender (%)				<0.001
Women	5378 (41.46)	11983 (44.72)	19142 (60.97)	
Men	7594 (58.54)	14813 (55.28)	12256 (39.03)	
Marital status (%)				<0.001
Living without partner	2698 (20.80)	4529 (16.90)	5446 (17.35)	
Living with partner	10274 (79.20)	22267 (83.10)	25952 (82.65)	
Smoking (%)				<0.001
Never or previous	11057 (85.24)	22931 (85.58)	28034 (89.29)	
Current	1915 (14.76)	3865 (14.42)	3364 (10.71)	
Drinking (%)				<0.001
Never	12015 (92.62)	24726 (92.27)	29232 (93.10)	
Occasionally	771 (5.94)	1674 (6.25)	1634 (5.20)	
Daily	186 (1.43)	396 (1.48)	532 (1.69)	
Physical activity (%)				<0.001
Never	10774 (83.06)	21974 (82.00)	25225 (80.34)	
Occasionally	1159 (8.93)	2249 (8.39)	2820 (8.98)	
Daily	1039 (8.01)	2573 (9.60)	3353 (10.68)	
WC	75.00 (70.00, 80.00)	80.00 (77.00, 85.00)	82.00 (76.00, 89.00)	<0.001
BMI	22.44 (20.90, 23.59)	23.44 (22.10, 24.97)	24.45 (22.86, 26.63)	<0.001
BRI	2.62 (2.22, 2.92)	3.30 (2.87, 3.71)	3.58 (2.84, 4.40)	<0.001
Time of follow-up	6.91 (5.03, 8.87)	8.01 (5.95, 8.93)	8.01 (6.00, 8.92)	<0.001

Data are presented, median (interquartile range), or number (percentage).

BMI, body mass index; WC, waist circumference; BRI, body roundness index.

Supplementary Table 4 Latent Class Growth Mixture models (LCGMM) results when excluding those participants with less than 3 years of follow-up

Nb. Latent classes	Polynomial degree	Log-Lik	BIC	% Participants per class	Mean posterior probabilities	Posterior probabilities>0.7 (%)
1	Linear	-376664.8	753396.3	100	n.a	n.a
	Quadratic	-371854.7	743820.6	100	n.a	n.a
	Cubic	-370565.2	741297.4	100	n.a	n.a
2	Linear	-375424.2	750959.7	49.04/50.96	0.70/0.81	54.79/69.84
	Quadratic	-368980.3	738127.5	46.62/53.38	0.73/0.85	63.43/76.63
	Cubic	-364706.8	729647.4	39.63/60.37	0.77/0.89	70.57/84.06
3	Linear	-374757.2	749670.1	72.56/9.86/17.58	0.77/0.67/0.71	74.68/40.09/51.56
	Quadratic	-367529	735280.6	18.16/43.73/38.1	0.76/0.86/0.67	63.70/79.25/44.99
	**Cubic**	**-366615.5**	**733531.5**	**46.87/46.24/6.89**	**0.71/0.82/0.77**	**60.52/71.16/62.70**
4	Linear	-374850.9	749902.2	19.13/46.51/9.54/24.83	0.74/0.61/0.62/0.53	60.34/25.83/25.35/0
	Quadratic	-366752.1	733782.4	4.25/26.28/39.21/30.26	0.77/0.70/0.84/0.63	65.07/56.77/75.50/32.47
	Cubic	-366155.3	732677.9	18.82/38.7/29.98/12.49	0.73/0.62/0.72/0.65	59.30/30.27/53.92/38.41

Supplementary Table 5 Cox regression analysis between BRI and all-cause mortality and cardiovascular mortality when excluding those participants with less than 3 years of follow-up

	Model 1			Model 2			Model 3		
	HR	95%CI	P	HR	95%CI	P	HR	95%CI	P
All-cause mortality									
Q1(2.03- 2.54)	1(Ref)			1(Ref)			1(Ref)		
Q2(2.82- 3.11)	0.89	0.84, 0.93	<0.001	0.98	0.93, 1.03	0.464	0.98	0.93, 1.03	0.369
Q3(3.36- 3.67)	0.80	0.76, 0.84	<0.001	0.91	0.86, 0.96	<0.001	0.91	0.86, 0.96	<0.001
Q4(4.09- 4.91)	0.89	0.84, 0.94	<0.001	0.96	0.91, 1.01	0.128	0.97	0.91, 1.01	0.135
P_{trend}			<0.001			0.030			0.035
Low-stable	1(Ref)			1(Ref)			1(Ref)		
Moderate-stable	1.14	1.09,1.19	<0.001	1.15	1.10, 1.20	<0.001	1.15	1.10, 1.20	<0.001
High-stable	1.99	1.89,2.09	<0.001	1.69	1.61, 1.78	<0.001	1.69	1.61, 1.78	<0.001
CVD mortality									
Q1(2.03- 2.54)	1(Ref)			1(Ref)			1(Ref)		
Q2(2.82- 3.11)	0.83	0.77, 0.89	<0.001	0.92	0.86, 0.99	0.034	0.92	0.85, 0.98	0.024
Q3(3.36- 3.67)	0.81	0.75, 0.87	<0.001	0.92	0.85, 0.99	0.027	0.92	0.85, 0.99	0.021
Q4(4.09- 4.91)	0.91	0.84, 0.98	0.009	0.98	0.91, 1.05	0.543	0.97	0.90, 1.05	0.485
P_{trend}			0.010			0.552			0.502
Low-stable	1(Ref)			1(Ref)			1(Ref)		
Moderate-stable	1.08	1.01, 1.15	0.018	1.10	1.03, 1.17	0.005	1.10	1.03, 1.17	0.004
High-stable	1.86	1.74, 2.00	<0.001	1.59	1.49, 1.71	<0.001	1.60	1.49, 1.71	<0.001

Data were showed by HR, 95% CI and P value.
Model 1: Unadjusted.
Model 2: Adjusted for age and gender.
Model 3: Adjusted for age, gender, smoking, alcohol consumption, and physical activity.
Abbreviations: Q1: 1st Quartiles; Q2: 2nd Quartiles; Q3: 3rd Quartiles; Q4: 4th Quartiles. Q1 to Q4 are the quartiles of BRI at baseline.

Supplementary Table 6 Latent Class Growth Mixture models (LCGMM) results of BMI

Nb. Latent classes	Polynomial degree	Log-Lik	BIC	% Participants per class	Mean posterior probabilities	Posterior probabilities>0.7 (%)
1	Linear	-631554.3	1263176	100	n.a	n.a
	Quadratic	-628676.7	1257465	100	n.a	n.a
	Cubic	-629016.9	1258201	100	n.a	n.a
2	Linear	-631554.3	1263209	46.72/53.28	0.51/0.49	0/0
	Quadratic	-628676.7	1257510	44.96/55.04	0.53/0.52	0/0
	Cubic	-628730.1	1257684	46.55/53.45	0.53/0.53	0.02/0
3	Linear	-629471.1	1259076	2.83/93.28/3.89	0.76/0.23/0.01	62.05/95.33/61.76
	Quadratic	-624393.7	1248989	2.51/92.35/5.14	0.85/0.95/0.75	79.73/95.68/57.87
	**Cubic**	**-619469.7**	**1239219**	**2.33/94.03/3.64**	**0.88/0.99/0.90**	**82.99/98.85/87.10**
4	Linear	-631544.7	1263257	19.53/3.8/69.25/7.42	0.30/0.30/0.32/0.30	0/0/0/0
	Quadratic	-628670.3	1257586	38.35/3.55/46.72/11.38	0.31/0.26/0.28/0.29	0/0/0/0
	Cubic	-622037.9	1244411	1.68/94.95/0/3.36	0.81/0.66/ n.a/ 0.83	69.45/ 15.40/ n.a/ 73.81

Supplementary Table 7 Latent Class Growth Mixture models (LCGMM) results of WC

Nb. Latent classes	Polynomial degree	Log-Lik	BIC	% Participants per class	Mean posterior probabilities	Posterior probabilities>0.7 (%
1	Linear	-976096.9	1952261	100	n.a	n.a
	Quadratic	-971786.4	1943685	100	n.a	n.a
	Cubic	-1e+09	2000000168	100	n.a	n.a
2	Linear	-976096.9	1952294	49.16/50.84	0.52/0.52	0/0
	Quadratic	-971786.4	1943729	51.75/ 48.25	0.51/0.51	0/0
	Cubic	-972258.3	1944740	48.73/51.27	0.62/0.61	17.58/16.89
3	Linear	-975535.2	1951204	94.32/0.83/4.85	0.91/0.65/0.69	93.18/33.96/44.19
	Quadratic	-971289.6	1942780	86.98/3.57/9.46	0.80/0.54/0.64	77.10/9.97/35.05
	**Cubic**	**-969699.4**	**1939678**	**84.09/4.94/10.97**	**0.90/0.73/0.75**	**88.91/53.13/58.54**
4	Linear	-976004.3	1952176	1.64/0/68.2/30.15	0.31/n.a/0.35/0.48	0/n.a/0/11.88
	Quadratic	-1e+09	2000000246	0/0/0/0/0	n.a/n.a/n.a/n.a	n.a/n.a/n.a/n.a
	Cubic	-969598.9	1939533	34.21/49.92/4.17/11.7	0.59/0.59/0.68/0.67	15.15/13.65/45.97/44.85

Supplementary Table **8** Cox regression analysis between trajectories of BMI and all-cause mortality and cardiovascular mortality

	Model 1			Model 2			Model 3		
	HR	95%CI	P	HR	95%CI	P	HR	95%CI	P
All-cause mortality									
Low-stable	1(Ref)			1(Ref)			1(Ref)		
Moderate-stable	1.01	0.92,1.11	0.828	0.98	0.89,1.08	0.664	0.98	0.89,1.08	0.711
High-stable	1.01	0.87,1.18	0.858	0.99	0.86,1.15	0.943	0.99	0.86,1.16	0.982
CVD mortality									
Low-stable	1(Ref)			1(Ref)			1(Ref)		
Moderate-stable	1.03	0.90, 1.19	0.627	1.01	0.87, 1.15	0.992	1.01	0.88, 1.15	0.942
High-stable	1.01	0.82, 1.25	0.917	0.99	0.80, 1.22	0.937	0.99	0.81, 1.23	0.987

Data were showed by HR, 95% CI and P value.
Model 1: Unadjusted.
Model 2: Adjusted for age and gender.
Model 3: Adjusted for age, gender, smoking, alcohol consumption, and physical activity.
BMI, body mass index.

Supplementary Table **9** Cox regression analysis between trajectories of WC and all-cause mortality and cardiovascular mortality

	Model 1			Model 2			Model 3		
	HR	95%CI	P	HR	95%CI	P	HR	95%CI	P
All-cause mortality									
Low-stable	1(Ref)			1(Ref)			1(Ref)		
Moderate-stable	0.98	0.90,1.07	0.685	0.99	0.91,1.08	0.799	0.99	0.91,1.08	0.813
High-stable	1.02	0.93,1.13	0.639	1.02	0.93,1.13	0.654	1.02	0.93,1.13	0.645
CVD mortality									
Low-stable	1(Ref)			1(Ref)			1(Ref)		
Moderate-stable	1.04	0.92,1.17	0.552	1.05	0.93,1.18	0.468	1.05	0.93,1.18	0.462
High-stable	1.08	0.94,1.24	0.277	1.08	0.94,1.24	0.285	1.08	0.94,1.24	0.280

Data were showed by HR, 95% CI and P value.
Model 1: Unadjusted.
Model 2: Adjusted for age and gender.
Model 3: Adjusted for age, gender, smoking, alcohol consumption, and physical activity.
WC, waist circumference.

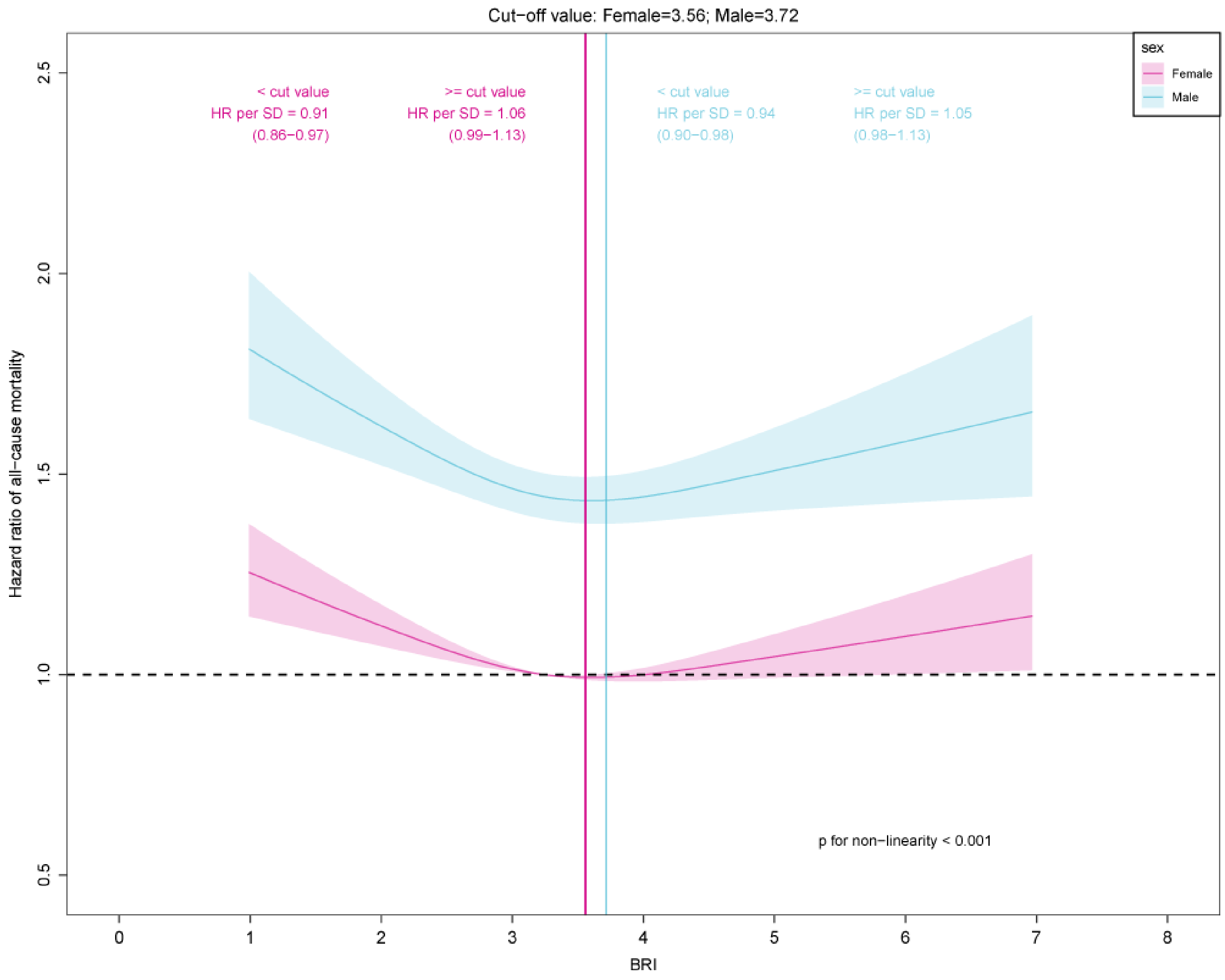

Supplementary Figure 1 Subgroup analysis by sex in restricted cubic spline plots of the relationship between BRI and all-cause mortality.

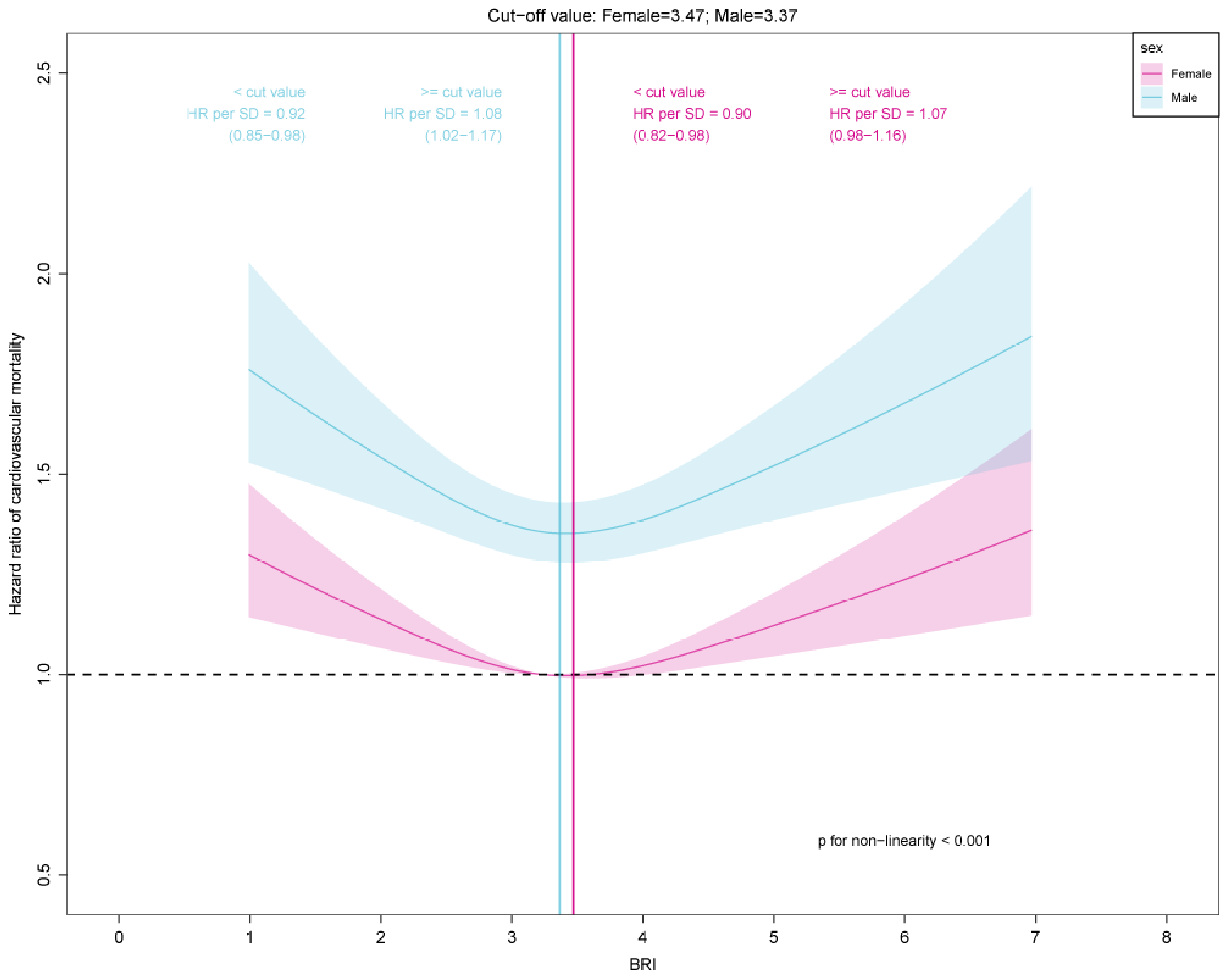

Supplementary Figure 2 Subgroup analysis by sex in restricted cubic spline plots of the relationship between BRI and CVD mortality.

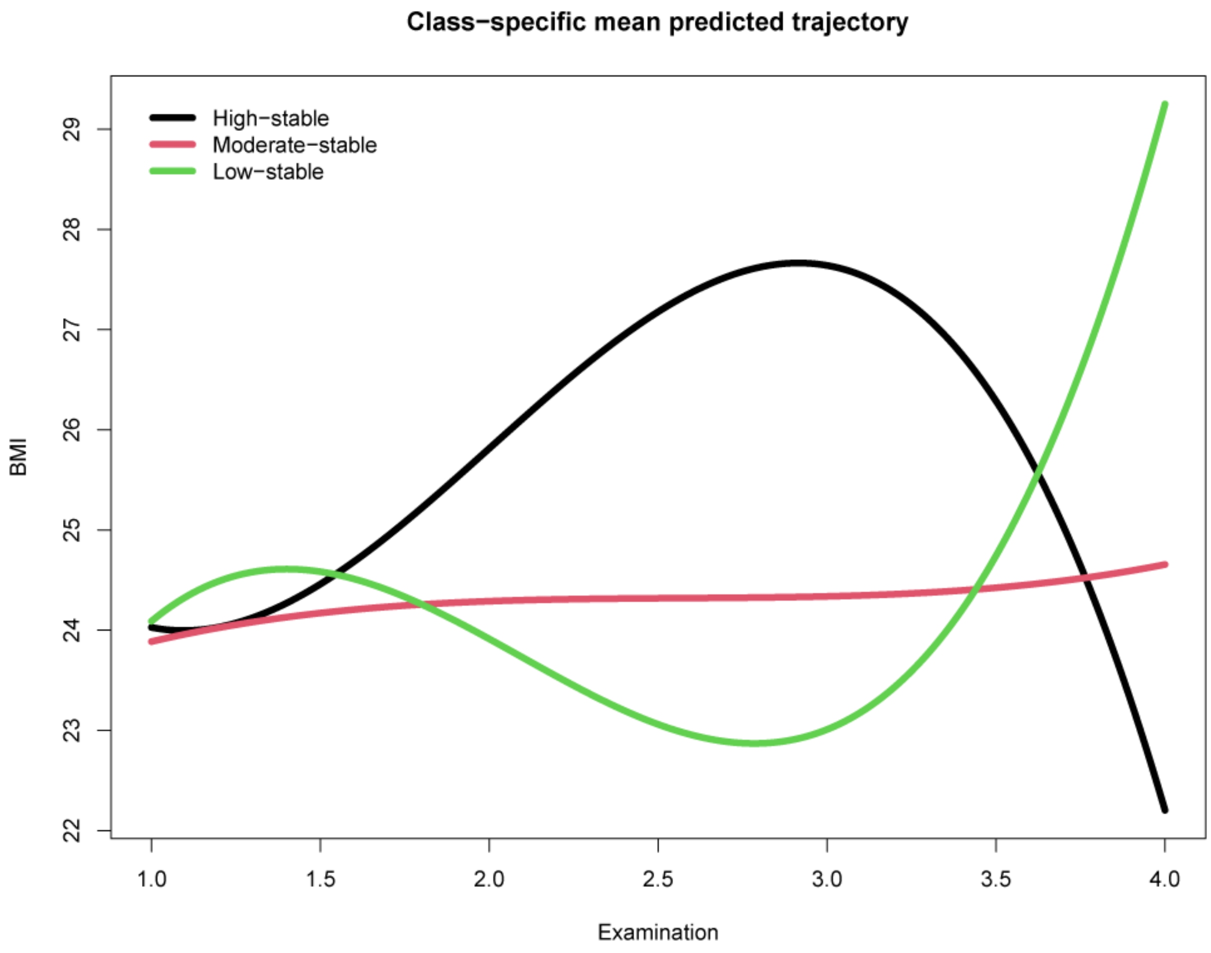

Supplementary Figure 3 Trajectories of body mass index from the first physical examination to the fourth.

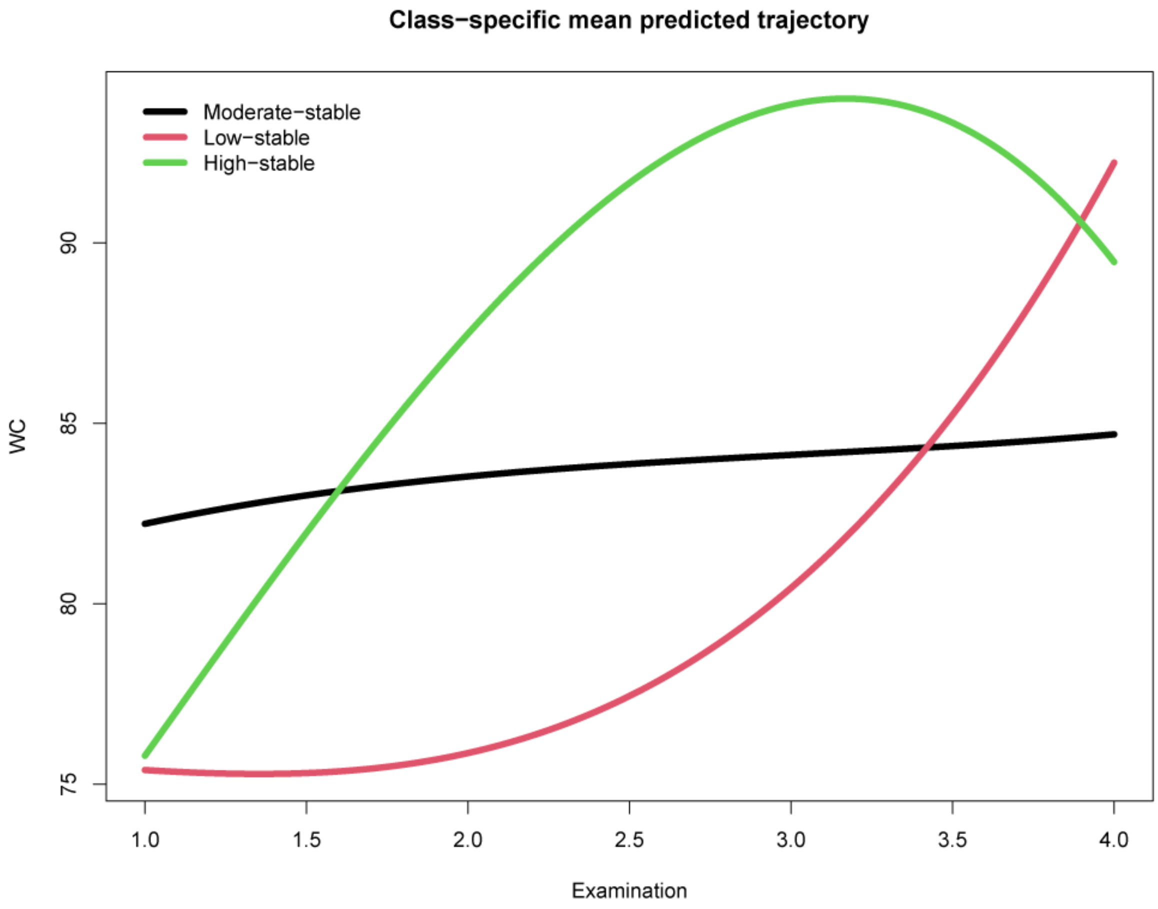

Supplementary Figure 4 Trajectories of waist circumference from the first physical examination to the fourth.
